# Sterilization of the Unfit
*Communicated to the Bristol Medico-Chirurgical Society, 13th May, 1931.


**Published:** 1931

**Authors:** H. C. Bristowe


### STERILIZATION OF THE UNFIT.*

BY

H. C. Bristowe, M.D.

---

The subject of sterilization has been so much in the public eye of late that it may be as well to consider whether there are likely to accrue any real benefits from adopting it as a method of dealing with a certain class of case. I do not propose to give my own views as to the utility of it, as (to tell the truth) they are still fluid; but rather is it my intention to give some of the arguments both for and against its adoption, and to try and gather what the general opinion of the profession in Bristol is.

This subject must be considered from the religious, legal, ethical, scientific and social aspects before any definite conclusion can be arrived at as to whether powers should be obtained to legalize such an operation.

The *religious* point of view need not detain us long. The Roman Church is, as one would expect, dead against it. The view taken by it is that we have no moral or religious right to interfere with the procreation of children, or in any way to prevent any man or woman becoming a parent. The one exception is that if it is necessary for the health of a man or woman that a diseased organ should be removed, which is incidentally one of the organs of generation it is permissible. But it must be for that definite

* Communicated to the Bristol Medico-Chirurgical Society, 13th May, 1931.

purpose, and not for the purpose of preventing procreation. The other churches have not spoken so definitely; indeed, judging from the findings of the Lambeth Conference it would appear that no objections would be raised by the Anglican Church as a whole, though this is probably not true of all its individual members. The attitude of the Churches must, however, be taken into account, if the profession or the public should ever apply to Parliament for the legalization of sterilization.

The *ethical* position is a more difficult one. It is to some extent linked up with the religious. Do we consider that we have a right to mutilate our fellow-creatures? Are we justified in subjecting them to the risks of operation? Do we think that we have a right to say to anyone: "Thou shalt not breed"? Have we the right to say to any woman: "Thou shalt not enjoy the joys of motherhood"? Have we any ethical right to say to any man: "Thou shalt not have the pleasures of a family life in a home of thine own"? Then is it wise to let loose a number of women, who are in no danger of motherhood, and who would possibly have no motives for chastity? Would they not be in great demand among men of a certain type, and thus become carriers of venereal disease? Also I would ask, in a more tentative way, would not sterilized men be in request among women of a poor moral type? I know that the Eugenic Society maintain that such men are not attractive to women; this may be so, but safety might well give an attraction which the man himself had not. It is suggested that efficient guardianship would reduce these risks to a minimum. I wonder how these efficient guardians would set about making their guardianship efficient ?

The *legal* position is very definite. The law says very plainly that sterilization, *quā* sterilization, is illegal, and that mutilation is an offence. The British Medical Association took the opinion of counsel on this question of legality. Sir Travers Humphreys gave his opinion in the following terms :

"I am clearly of opinion that any medical man who performs the operation described upon a defective within the meaning of that term, as defined within the Mental Deficiency Act 1913, would in the present state of the law be acting illegally and without any legal justification. I assume the consent of both parents and the excellence of the motives of all concerned, but the fact remains that the operation of sterilization involves an assault upon and the wounding of the person operated upon. The only legal justification for such an action in regard to a person who, either from extreme youth or old age, or from any other cause such as mental weakness, is incapable of giving a reasoned consent, would be that the operation was necessary to the health or well-being of the patient. I do not gather from the case that it is contended that sterilization would improve the condition, physical or mental, of the defective.

"The legal risks involved in such an operation would attach equally to all persons concerned—the doctor who performs it, and the parent or guardian who requested or sanctioned it.

"The doctor would have, in my opinion, no answer to an indictment for the offence of unlawful wounding contrary to Section 20 of the Offences Against the Persons Act 1861."

This is only a portion of the written opinion, but the rest strengthens and supports what I have extracted from it. As to the question of voluntary sterilization, Lord Riddell is of opinion that this would be equally illegal. Such, then, is the legal position of sterilization as the law stands at present.

I now come to the *social* side. It is of every bit as much importance as the scientific side; in fact, it may well be that the social is the most important aspect of the whole question. There cannot be any doubt that incidental sterilization, such as may be necessary for the removal of disease or for the saving of life, is not only justifiable but imperative. There is, however, another class of case, where the answer is not so clear. Are we justified in sterilizing a woman because her mental health would be endangered by pregnancy? If a woman has suffered from mental disease after each confinement, would sterilization be a justifiable operation? I feel inclined here to make an exception to my intention of not expressing my own opinion. Such a course seems to me to be quite a proper one, since every mental attack following each successive parturition leaves the woman at a lower mental level, until eventually there is a risk of complete dementia. No doubt abstinence is the right course. Failing that, contraceptives might be employed, but even here there is a possibility of accidents happening, which may endanger the sanity of the patients.

It has been urged that sterilization of defectives would effect a great saving of expenditure by local authorities, in that there would no longer be any necessity to erect and maintain institutions for these cases. Here there is a grave danger. If we are to have sterilization with that idea in view, we shall find ourselves in a predicament. We should still have with us a considerable number of persons at large who are now safe in public institutions—safe as regards not only their own but also the public safety. Many of these persons, if not almost all who are already in institutions, are quite unable to earn their own living or to take proper care of themselves, and institutional treatment would still be required for them.

I have already discussed the moral, or perhaps I should say the immoral, side of the question under the ethical aspects. And we must admit that this has a social as well as an ethical side. It seems that it is as objectionable from the social as from the ethical. We do not want to do anything which will add to the natural immorality of a certain section of the community.

We now come to the *scientific* side of the question. Sterilization has been chiefly pressed for as a means of preventing, or at least limiting, the number of mental defectives, which is at the present time stated to be on the increase. I do not for a moment say that it is not on the increase, I am not in a position to make any definite statement on this subject. It is argued that if mental defectives were sterilized, mental deficiency, even if not stamped out, would at least be greatly reduced.

There are, however, several difficulties which have to be overcome. What classes of defectives is it suggested should be sterilized? If the answer is "all," then we may ask what is the use of sterilizing an idiot who does not breed ? It must remain necessary that he should be dealt with either in an institution or under very efficient guardianship, since according to the statutory definition he is a person who is unable to protect himself against common physical dangers. He is able to do nothing for himself, not even capable of defending himself against an ordinary blow. He cannot feed himself. Next in order we have the various grades of imbeciles. These are very unlikely to breed, and cannot read, write or understand the value of money. They are quite unable to provide themselves with the ordinary necessaries of life, and must be detained in an institution for their own protection, if there is not very efficient guardianship. The lower grade imbecile cannot fend for himself, he is often vicious, and unfit to be at large. We now come to the higher grades—the mental defective, and it must be for these and these alone that sterilization can be asked for. Again, at what age is it suggested that this operation should be performed ?

It must be remembered that in childhood there has not been complete development of the mind any more than there has been of the body, and that at puberty a mental defective may either show signs of rapid deterioration, when sterilization would be a work of supererogation, as institutional treatment, or effective guardianship, is essential for his own safety. On the other hand, at that age there may begin a distinct improvement in his mental activities, and in such cases sterilization would be unjustifiable. Then, again, we shall have to decide on the grade of deficiency that we consider would justify such an operation. How are we to arrive at that ? What may appear to be defective in one grade of society might possibly pass as normal, or even above normal, in others. We must admit that the surroundings in which a mental defective is brought up may to no small extent affect his mental state. Herd,[1] in his book, says: "When native intelligence is not fostered by a normal contact with the outer world of persons and events, both it and specific capacities do not develop as they should normally, and certain capacities may even atrophy to a greater or less degree." Also:[2] "The mental ratio of a normal child whose education, either formal or natural, is seriously interfered with is liable to fall, and usually does fall."

Again, quoting from Herd's work: "Dr. Lewis, in his report on his own survey, says that for every defective there are two or more others only slightly higher in mental and educational capacity, and at a mental welfare conference, held just after the issue of the report, he stated that for every defective there were ten others subnormal, that is, enough to make them relatively and in various degrees incapable of coping with the elementary school curriculum."

It seems quite obvious from the above quotations that the question of what defectives shall be sterilized and what shall not be is by no means an easy one. Nor are we in a position to say to what extent, if any, are these various degrees of mental deficiency hereditary. They may all be hereditary. If so, is it proposed that all persons showing some incapacity of imbibing knowledge or exercising wisdom and prudence are to be subject to sterilization? I admit that the compulsory sterilization of all such, as well as all physical degenerates, is certainly Utopian, but it can hardly be considered as practical in the present time.

Once more, are we to sterilize those who are educationally defective, but who are quite capable of earning their own living. Or are we to advise it only for those who are socially defective, that is, those who, though showing some power of imbibing knowledge, are yet incapable of adjusting themselves to modern civilized conditions, including the erratic genius who has much wit and power in some directions, and yet is quite unable to adjust himself to his surroundings and so earn his own bread ?

But supposing we can arrive at some definite standard, it does seem to me that it will have to be a very low standard. What, may I ask, is the standard, not of education, but of mentality of the greater proportion of the inhabitants of these islands of ours ? Is it not a very low one ? Are we to take our standard from the universities, from our professional classes, from our public schools ? Or are we to take them from the elementary schools ? It seems to me that some standard, low or high, must be taken, and the same standard adopted for all classes. I refer you once more to the quotations I have taken from Dr. Herd's book. It does appear that what may be taken as a high standard for some classes would be a very low one for others.

Those who advocate sterilization maintain that the result of it would be to diminish the incidence of mental deficiency, and believe that in the course of years this incidence would be much reduced. This argument is based on a belief that mental deficiency is hereditary on Mendelian principles. It is for these advocates to produce some sort of proof that their theories are correct. At present there is very little evidence that can be produced. I do not think that I can do better than quote from a brochure published by the Eugenics Society,[3] a very moderately-written brochure in favour of sterilization: "Some think that defectiveness is transmitted along Mendelian lines by a single recessive factor, or by a number of different factors. Others, such as Myerson, Devine and Tredgold, think that defectiveness is largely produced by toxins acting upon the spermatozoon or ovum or early embryo according to the so-called law of blastophoria."

Tredgold,[4] in a note published in *Mental Welfare*, says:—

"It is now generally recognized that the majority of cases are a result of 'inheritance.' It has consequently, not unnaturally, been assumed by those who have no great experience of defectives, and who are unacquainted with the subject of heredity, that the mentally defective child must necessarily be the offspring of mentally defective parents. It is of the utmost importance to realize that this is not so, and that the defect of a child may be due to inheritance without its parents being similarly defective. The explanation of this paradox lies in the nature of inheritance of these cases. Although some writers, chiefly in America, have attempted to prove that mental deficiency is due to the absence in the ancestral germ cells of certain definite constituents, and that transmission is in accordance with the laws formulated by Mendel, these attempts have been entirely unsuccessful. On the contrary, there is every reason to conclude that mental defect is not due to the absence from the germ of a definite factor or series of factors ; but that it is the expression of an impairment of developmental potentiality. In other words, it is due to a qualitative rather than a quantitative change ; this change being in the nature of a germinal vitiation. There is reason to think, however, that the amount of this vitiation varies not only in the germ cells of different individuals of the same stock, but also in the germ cells of the same individual. Moreover, it is highly probable that even with vitiated germ cells the condition of the offspring resulting therefrom will be to some extent influenced by the nature of the ante-natal environment. The result is that, although the majority of mental defectives come of families whose germ cells have undergone vitiation, this change shows itself in many other ways than mental defect. In some individuals it may be manifest as dementia precox or other forms of dementia ; in others as certain forms of insanity or mental instability. . . . Nevertheless, it is probable that all these classes of persons are 'carriers' of mental defect, and that a chance combination of adverse circumstances may at any time so embarrass the development of the impaired germ cells as to result in a mentally deficient offspring."

Again I may ask the question : Is mental deficiency the result of germ cells which are defective primarily or secondarily ? Is the impairment of the germ cell due to defects inherited from one or both parents, or is it due to some damage to the germ cell after impregnation, or in the course of development ? We do know that some diseases occurring in the developing brain will cause mental defects, as, for instance, encephalitis lethargica. If mental defects are chiefly due to inherited defects, then sterilization should, theoretically at any rate, be of use in limiting the number of defectives in the future. If, however, mental deficiency is due to injury of the germ cells, or to developmental errors, then sterilization would be of no value. Sterilization is legal and has been practised in some of the American states, and the results, so far, have not been very encouraging. Shrubsall[5] states that "several lines of inquiry seem to show that only 5 per cent. of defective children are the offspring of defective parents, though even in these the parents were rarely of a mental status that would have permitted of certification even had they been subject to be dealt with. The proportion of those parents who were defective in one investigation was less than those who were called superior." Some authorities consider that there are no forms of mental deficiency which can be considered hereditary.

If, then, sterilization of the mentally unfit is to be approved, it seems clear that we must obtain much more information than we have at present. In fact, there are such differences among those who are best able to give considered judgments on this subject that we must at present agree that we are not in possession of sufficient evidence to form any opinion of value. We require much information before we can ask for legislation to give effect to sterilization. What, then, are the conclusions at which we must necessarily arrive? First, we require and must obtain more knowledge as to the cause of mental deficiency than we have at present. Next, it must be clearly pointed out to all the authorities that at its best sterilization can be of no use as an alternative to segregation, that no sterilization will do away with the need of institutional treatment and efficient guardianship, and that whatever range of sterilization may be permissible in the future, the amount of money spent on the segregation of mental defectives cannot be reduced, but will have to be increased. This will be increasingly clear when it is fully understood that a large percentage of recidivists are of the mentally defective classes—persons who are unable from mental defect to lead useful lives outside institutions, and almost perforce take to criminal lives.

Of course, if we choose to adopt quite a different attitude (as we are at liberty to do) we may say quite logically: We consider that we should permit and try to enforce sterilization on all degenerates whether mental or physical, and thus prevent the breeding of the large masses that at present bring numbers of undersized, under-intelligent and criminal persons into this world only to be kept at the expense of those who are not degenerates. For there can be but little doubt that at present we are helping to bring about a course that is contrary to nature, the survival of the unfit. This would mean such a wholesale sterilization that the public would not for one moment hear of it, as so many would be honestly wondering if their own turn would not be coming next. It is not within the range of practical politics.

I will close by reading to you the resolution passed by the Executive of the Central Association for Mental Welfare in London, a resolution with which I agree :—

"That they are of opinion that there are certain selected cases of mental defect in which sterilization might be an appropriate and desirable procedure; if there is any doubt as to the legality of the operation in such cases, the Committee are of opinion that the Association should favour legislation to admit of such selective sterilization, provided that adequate safeguards can be devised to prevent its improper use and to restrict its performance in such cases.

"That in regard to the question of the wholesale sterilization of defectives as a means of greatly reducing the incidence of mental deficiency, the Medical Committee know of no evidence in support of this, and see no reason for altering their opinion that such would be attended with comparatively insignificant results."

### Discussion.

Dr. R. J. A. Berry, in opening the discussion, congratulated the Society on having three members on the B.M.A. Committee for Mental Deficiency. He stated that he had been very favourably impressed by the way in which the results of sterilization in California had been stated in *Sterilization for Human Betterment.* He did not think that sterilization would stamp out mental deficiency, although he had no doubt of the strong hereditary factor in its causation, and he thought sterilization would do little to prevent feeble-mindedness. This question is one of the greatest problems of the modern state, and all methods so far employed to check its increase had failed. He would like to see the medical profession given the right to perform sterilization under conditions determined by the State. He felt that females were more dangerous than males when not under control, especially those of high or medium grades of mental deficiency.

Mr. C. F. WALTERS considered that the question was not, "Dare we perform sterilization ?" but rather "Dare we not ?" He thought that the first step was to educate the medical profession to a knowledge of the influence played by heredity, by means of discussions in all medical societies, and then the lay public should be similarly educated. In his opinion the first duty of medicine was to the race, and not to the individual.

Dr. J. R. CHARLES said that in his capacity of medical visitor to Stoke Park Colony he found that nearly every case of mental deficiency that he saw gave a strong family history. He found the question of letting patients free from control a very responsible one, and he did not think that sterilization would do away with the need of institutional treatment, since the liberation of large numbers of sterilized mental defectives would soon result in a large increase in venereal disease.

Dr. J. O. SYMES considered that the certification of mild degrees of mental deficiency was very difficult, and he thought that if sterilization was involved in certification it would make the certifying officers chary of labelling a child mentally defective unless the case was extreme. Moreover, these extreme cases needed care and supervision, so that in these cases sterilization was superfluous. He did not agree that there was a large element of heredity in the etiology of mental deficiency, but thought that a lot of the trouble was environmental in origin, many of the children being illegitimate and brought up in institutions where they lived very difficult and cramped lives. In better class families he found no hereditary history of the condition, and the cases were regarded as freaks in the family. Why should mental deficiency be singled out for sterilization any more than any other physical defect ? Finally, he considered the great objection to sterilization was the sentimental one.

Dr. R. G. GORDON regarded voluntary sterilization as a very foolish idea, as the only result would be an increase in venereal disease. If the profession considered sterilization justifiable, he urged them to go all out for it, and to drop such half measures as voluntary schemes. He personally did not approve of it, as he did not see how it could in any way lessen the need for institutional treatment.

Dr. H. H. CARLETON, while agreeing with the hereditary factor in mental deficiency, thought that this factor was probably a Mendelian recessive, and that the original delinquent was now far back in the common stock ; so that he regarded sterilization at the present time as merely pruning the periphery.

Dr. A. G. MORISON quoted figures showing that in mentally defective families only 33 per cent. of the children were mentally deficient, so that in order to prevent 100 cases of mental deficiency 200 normal children would have to be sacrificed. He thought that sterilization would be very slow in its effects.

Dr. W. R. DAWSON found a larger incidence of mental deficiency in the lower grade schools, and he thought that while the majority of cases were hereditary all were not. Sterilization might prevent the birth of a certain number but by no means all, and in any case institutional treatment would be necessary for life. He considered that the Scotch method of boarding out these cases merited consideration.

Dr. A. M. B. Milner thought that possibly creative evolution, as taught by Bernard Shaw, might counteract the Mendelian factor in the inheritance of mental deficiency.

Dr. F. Bodman stated that the country was grossly over-populated, and that better than sterilization would be the discovery of a fool-proof method of contraception.

Dr. Preston King did not doubt the hereditary element in the causation of mental deficiency, and thought that breeding from defective stocks should be avoided. He suggested that sterilization should apply to all cases well enough to be discharged from institutions.

Dr. Bristowe, in reply, agreed that sterilization could never replace segregation.

### REFERENCES.

[1] Herd, H., *The Diagnosis of Mental Deficiency*, London, 1930.

[2] Herd, H., *The Diagnosis of Mental Deficiency*, London, 1930.

[3] *Eugenic Sterilisation*, second edition, The Eugenic Society.

[4] Tredgold, A. F., *Mental Welfare*, xi. 9–14, 1930.

[5] Shrubsall, F. C., " Conference on Mental Welfare," Report of Session, 12th December, 1930.

